# HTLV-1 Tax peptide-carrying polyion complex nanoparticles induce potent cellular immunity in mice

**DOI:** 10.1186/1742-4690-11-S1-P63

**Published:** 2014-01-07

**Authors:** Tomofumi Uto, Takami Akagi, Mitsuru Akashi, Masanori Baba

**Affiliations:** 1 Graduate School of Engineering, Osaka University, Suita, Osaka, Japan; 2 Center for Chronic Viral Diseases, Graduate School of Medical and Dental Sciences, Kagoshima University, Sakuragaoka, Kagoshima, Japan; 3 JST-CREST, Shibuya-ku, Tokyo, Japan

## 

Development of safe and effective vaccines is important for controlling a variety of infectious diseases, including retroviral infections. The induction of cytotoxic T lymphocytes (CTLs) is a promising strategy for elimination of infected cells. Polyion complex (PIC) nanoparticles have been created using anionic biodegradable poly(γ-glutamic acid) (γ-PGA) and cationic protamine. Amphiphilic graft copolymers, consisting of γ-PGA and l-phenylalanine (l-Phe) hydrophobic side chain, were synthesized by grafting l-Phe to γ-PGA. The PIC nanoparticles were prepared by mixing the graft copolymers with protamine in phosphate buffered saline. In this study, antigen peptide-carrying PIC nanoparticles were examined for their effect on the induction of antigen-specific cellular immunity in mice. The antigen-specific CTL response was evaluated by intracellular cytokine staining and IFN-γ ELISPOT assay. The immunization with PIC nanoparticles carrying HTLV-1 Tax peptide could induce the expansion of Tax-specific CD8^+^ T cells. In contrast, no such induction of the antigen-specific CD8^+^ T cells was observed, when mice were immunized with the peptide alone or peptide plus an aluminum adjuvant. These results suggest that the Tax peptide-carrying PIC nanoparticles are capable of inducing cellular immune responses and may have potential as an effective vaccine adjuvant for anti-HTLV-1 vaccines.

